# Porous construction and surface modification of titanium-based materials for osteogenesis: A review

**DOI:** 10.3389/fbioe.2022.973297

**Published:** 2022-08-25

**Authors:** Rui Wang, Shilei Ni, Li Ma, Meihua Li

**Affiliations:** ^1^ Department of Stomatology, The Second Hospital of Jilin University, Changchun, China; ^2^ Department of Plastic and Aesthetic Surgery, Hospital of Stomatology, Jilin University, Changchun, China; ^3^ Department of Fever Clinic, The Second Hospital of Jilin University, Changchun, China

**Keywords:** surface modification, Micro-arc oxidation (MAO), porous, implants, osteogenesis, Ti-based materials

## Abstract

Titanium and titanium alloy implants are essential for bone tissue regeneration engineering. The current trend is toward the manufacture of implants from materials that mimic the structure, composition and elasticity of bones. Titanium and titanium alloy implants, the most common materials for implants, can be used as a bone conduction material but cannot promote osteogenesis. In clinical practice, there is a high demand for implant surfaces that stimulate bone formation and accelerate bone binding, thus shortening the implantation-to-loading time and enhancing implantation success. To avoid stress shielding, the elastic modulus of porous titanium and titanium alloy implants must match that of bone. Micro-arc oxidation technology has been utilized to increase the surface activity and build a somewhat hard coating on porous titanium and titanium alloy implants. More recently, a growing number of researchers have combined micro-arc oxidation with hydrothermal, ultrasonic, and laser treatments, coatings that inhibit bacterial growth, and acid etching with sand blasting methods to improve bonding to bone. This paper summarizes the reaction at the interface between bone and implant material, the porous design principle of scaffold material, MAO technology and the combination of MAO with other technologies in the field of porous titanium and titanium alloys to encourage their application in the development of medical implants.

## Introduction

With the growth of the aging population, titanium and titanium alloy materials have become increasingly widely used in implants. Titanium and titanium alloys are frequently utilized in biomedical implants due to their biocompatibility, strong mechanical qualities, and corrosion resistance (Geetha et al., 2009; Singh et al., 2013; Liu et al., 2017; Takizawa et al., 2018; Zhang and Chen, 2019; Chen L. Y. et al., 2020). However, because the elastic modulus of metal is substantially larger than that of bone, this results in a “stress shielding” effect that prevents the necessary stress from reaching the neighboring bone matrix, which eventually causes local osteoclast production and implant loss (Engh et al., 2003; Niinomi and Nakai, 2011; Manoj et al., 2018; Raffa et al., 2021). Bone is made of cancellous and cortical components. Bone density gradually increases from the inner cancellous bone to the outer cortical bone, revealing uneven and porous structure (Hadjidakis and Androulakis, 2006; Currey, 2011; Chen H. et al., 2020). By altering the size and structure of pores in implants, bone-like mechanical characteristics can be achieved (Engh et al., 2003; Niinomi and Nakai, 2011; Manoj et al., 2018; Raffa et al., 2021). In addition to assisting cell attachment, growth, division and differentiation, biocompatible metal implants should also facilitate the movement of nutrients and metabolic wastes (Ryan et al., 2008; Koolen et al., 2020; Lv et al., 2021; Rana et al., 2021). The traditional methods for manufacturing porous implant materials include powder metallurgy (Ryan et al., 2008; Nicoara et al., 2016; Rodriguez-Contreras et al., 2021), freeze drying (Sachlos and Czernuszka, 2003; Murphy et al., 2010), gas foaming (Sachlos and Czernuszka, 2003), spark plasma sintering (Annur et al., 2021), and open-pore titanium foam (Imwinkelried, 2007). However, the abovementioned porous materials have characteristics such as small pores, uneven pore distributions, poor permeability or numerous micropores within the pore wall structure that hinder their applications as biomaterials (Ryan et al., 2008; Koolen et al., 2020; Lv et al., 2021). The current design of materials for implants focuses on their elastic modulus, compressive strength, and biocompatibility (Chen H. et al., 2020; Dziaduszewska and Zielinski, 2021).

Because of the potential of 3D printing technology to create individualized and tailored products, its use in orthopedics and dentistry has expanded in recent years (Jing et al., 2020). Additive manufacturing (AM) technology allows the manufacture of implants that have regulated structures, shapes and properties. It is possible to directly design models with complex 3D hierarchical structures, which offers a new option for the preparation of porous implants (Huang and Schmid, 2018; Yu et al., 2018; Kim et al., 2020; Dziaduszewska and Zielinski, 2021; Khorsandi et al., 2021; Salmi, 2021). Ti6Al4V is the titanium alloy that is most frequently employed for 3D printing. Since Brånemark’s (1983) discovery of osseointegration between titanium and bone tissue, titanium and titanium alloy-based screw-type implants and orthopedic implant materials have been widely utilized in dentistry and orthopedic medicine.

Titanium and its alloys cannot bond well with bone due to biological surface inertia, and this affects implant success. Aseptic loosening and bacterial infection of implants are significant concerns in orthopedics and dentistry (Wang et al., 2020; Yin et al., 2022). Biofunctionalization to alter the morphology, structure, elements, and crystal phase composition endows the surfaces of titanium implants with biological activity, promotes their integration with bone tissue, and enhances their mechanical and antibacterial properties; it is the key to expanding the application of titanium implants (Zhang B. et al., 2018, 2020; Wang H. et al., 2021; Fazel et al., 2021). It is more challenging to modify the surfaces of porous implants than nonporous implants due to their unique shape.

**FIGURE 1:**
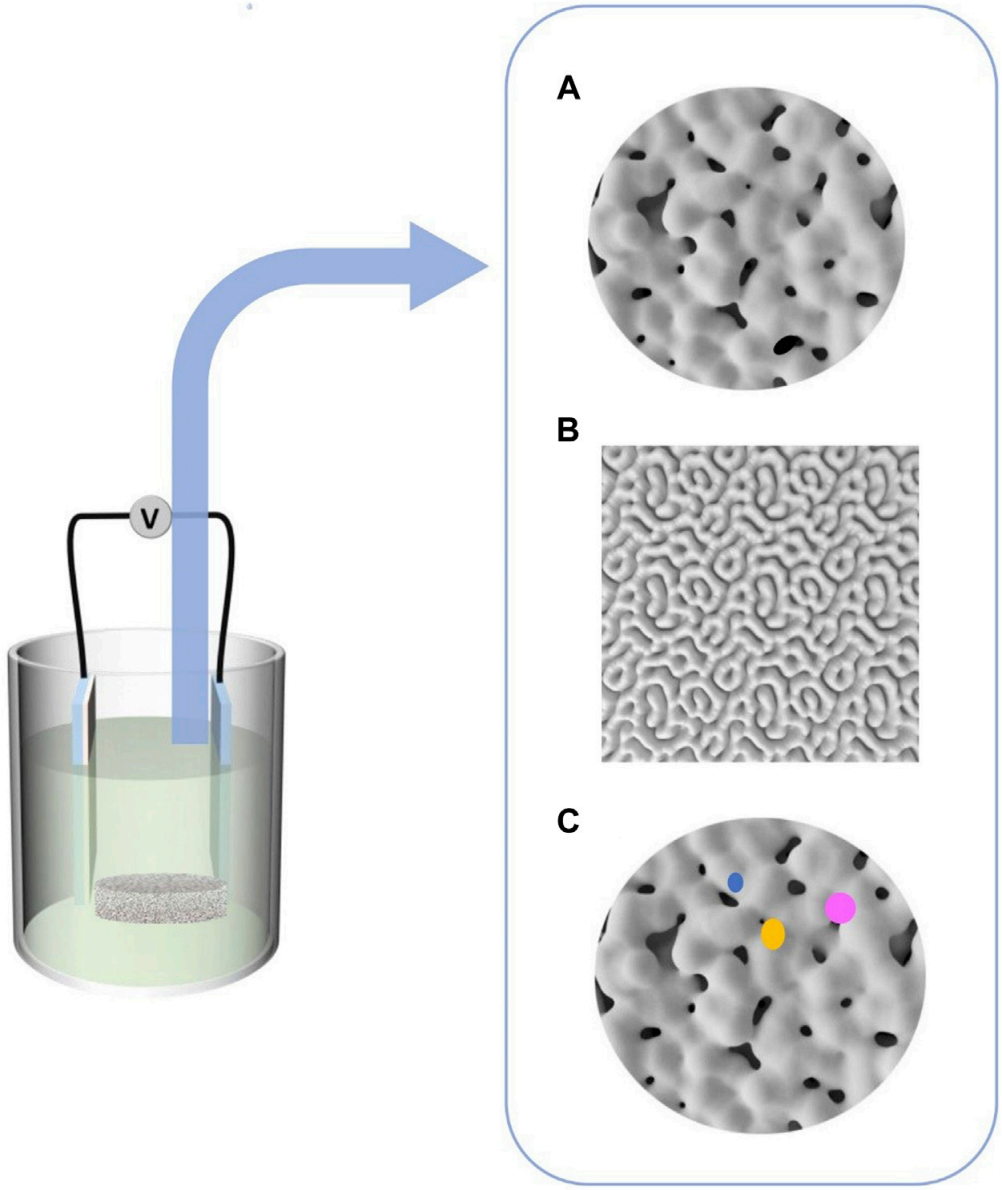
Different coating morphologies were formed by micro-arc oxidation, **(A)** Micropores of unequal size, **(B)** Cortex-like structure, **(C)** Bioactive ions coatings developed.

Micro-arc oxidation (MAO) technology has gradually gained popularity as an implant surface modification method because it is a convenient and efficient way to dope materials with active ingredients, change the morphology of the implant surface, and greatly enhance the biological activity of titanium-based implants. Under the influence of the immediate high temperature and high pressure produced by arc discharge, MAO, also known as plasma electrolytic oxidation (PEO) and anode spark deposition (ASD), is utilized to generate oxide coatings with complicated geometries on the surface of valve metals (Wang C. et al., 2018). This approach can incorporate bioactive electrolyte components such as calcium and phosphate into the coating. Calcium phosphate (CaP) coatings, particularly hydroxyapatite (HA) coatings, can facilitate early and rapid osseointegration. The bioactivity of the coating is determined by its physicochemical characteristics, including roughness, porosity, phase, elemental composition, and adhesive strength (Terleeva et al., 2010) (Figure 1)**.** In recent years, scientists have tried to combine MAO with other surface treatment technologies and achieve better bone bonding effect. Biocompatible implant materials must have appropriate size, shape, porosity, morphology, surface composition and protective coating. This paper reviews the reaction at the interface between bone and implant material, the porous design principle of scaffold material, MAO technology and the combination of MAO with other technologies in the field of porous titanium and titanium alloys to support their application in orthopedics and oral implants.

## Interface response between bone and implant material following implant placement

Osteoinduction, osteoconduction and osseointegration are connected to the process of bone regeneration following injury. Osteoinduction refers to inducing the development of new bone around the implant. Osteoinduction refers to the stimulation of primitive, undifferentiated and pluripotent stem cells to differentiate into osteoblast lines (Albrektsson, 2001). Osteoconduction is a term commonly used in the context of implants. Allowing bone to grow on the surface or inward into pores, channels, or tubes in implant is referred to as osteoconduction (Albrektsson, 2001). Osteointegration is the term used to describe the direct structural and functional link between the surface holding the implant and the organized living bone (Mavrogenis et al., 2009). In complex dynamic environment, the implant-tissue interface is characterized by the mechanical and biological interaction of the implant with the surrounding tissues. To establish long-term stability of the implant-tissue contact, the ultimate goal is to fully integrate the implant with tissues (Grzeskowiak et al., 2020).

The majority of the bone that is newly produced after implant insertion is formed by osteoinduction, which is a typical component of bone healing. Implantation of materials damage tissues during surgery, and bleeding is the first and most critical phase in the healing process (Grzeskowiak et al., 2020). After migrating from the capillary to the surface of the implant material, several blood components, including red blood cells, platelets and immune system cells, such as polymorphonuclear leukocytes and monocytes, are activated, releasing cytokines and other growth factors at the site of the injury (Mavrogenis et al., 2009). Hemostasis and coagulation occur 1 h after implantation, followed by granulation tissue formation within 2 h (Overmann et al., 2020). Bone formation requires several different kinds of bone growth factors, including bone morphogenetic protein (BMP), insulin-like growth factors (IGF I and II), fibroblast growth factor (FGF), transforming growth factor-β (TGF-β) and platelet-derived growth factor (PDGF). BMP is naturally released during trauma or bone reconstruction and is a known inducer. However, it is believed that nonintrinsic factors, such as stress or other forms of applied electrical impulses, can directly or indirectly influence osteoinduction (Albrektsson, 2001).

Bone growth at the implant’s surface is dependent on the functions of differentiated bone cells. These cells may originate from preexisting osteoblasts triggered by trauma or mesenchymal cells recruited through osteoinduction. Therefore, in practice, osteoconduction is heavily reliant on preceding osteoinduction. The proliferation of differentiated bone cells at the interface between implanted materials and natural bone is necessary for normal bone formation. Osteoconduction in implants is affected by bone regeneration conditions, biomaterials used and their reactions (Yao et al., 2013, 2021; Li et al., 2017). The surface properties of implant materials are crucial to their design and medical use since they are the initial point of interaction with the environment (Wu et al., 2014). For the implant to achieve long-term stability, its ultimate goal is to integrate with the surrounding tissue (Grzeskowiak et al., 2020). Several biological processes, such as the formation of hematomas, the growth of mesenchymal tissue, the production of intramembranous (woven) bone, and the construction of lamellar bone, have been found to occur after the implantation of implant material (Grzeskowiak et al., 2020). The most critical part of osteoconduction is the chain reaction on the surface of the implant, which induces osteoblast migration by activating platelets (Davies, 2003). When activated, platelets and inflammatory cells release chemical attractants, attracting fibroblasts to the site and forming fibrin matrix that promotes bone growth. Then, the fibrin matrix on the surface of the implant gradually changes into osteoid matrix, and its structure is similar to that of the bone cement line and bone plate with weakly mineralized osteoid tissue. As osteoblasts produce and secrete collagen to form calcified layers, osteoid tissues on the surface of implants slowly calcify. In the loading stage, mineralized osteoid tissue is transformed into braided bone, which fills the gap between the host bone and the implant but also helps to maintain its structural integrity (Grzeskowiak et al., 2020).

The third stage is osseointegration, which Brånemark et al. (2001) originally described. Albrektsson (2001) first defined direct touch as typical in any bone healing process. Three months after implantation, the woven bone gradually develops into dense lamellar bone. Osteoblasts form bone-like tissue and new bone trabeculae, which remodel into lamellar bone when in contact with the implant surface (osseointegration). When a thin layer of bone (comprising osteoblasts, osteoclasts, mesenchymal stem cells, lymphocytes, and blood vessels) surrounds the implant, the bone around the implant reaches its highest degree of tissue and mechanical qualities, and osseointegration of the implant is complete (Junker et al., 2009).

### Osteogenic Implant Material Design Requirements

Traditional implant design is usually based on statistical averages of the population, which results in a series of implant products with precise sizes. However, for patients whose bones have large defects or irregular shapes, traditional implants cannot be modified and customized according to individual needs. With the emergence of 3D printing technology, precision medicine can now be practiced (Lee et al., 2005). 3D printing can directly generate complex 3D layered structures from computational models (Bayata and Yildiz, 2020), which provides a new way to prepare porous implants (Van Bael et al., 2012; Taniguchi et al., 2016; Prasad et al., 2017; Zhang B. et al., 2018). The effective contact area controls how the implant material interacts with neighboring tissues when the implant surface directly contacts bone tissue. Implant structure can increase the contact area, which affects bone development at the junction between bone and implant. The change in the macrostructure of porous titanium alters its biocompatibility. Therefore, many studies have been devoted to fabricating porous Ti structures on implant materials, and their results have proven that bone development in porous structures occurred (Yang et al., 2017).

However, the implant materials industry has been focusing on establishing sufficient bone-implant contact, enhancing the biological activity of implant materials, and realizing the early function and long-term performance stability of implant materials (Jing et al., 2020). At present, there is no consensus on the structural requirements of ideal porous Ti6Al4V materials and no straightforward method to achieve high pore size uniformity. Therefore, the application of 3D printing technology to research and fabricate uniform pore structures with ideal characteristics and enhance their surface biological activity will provide a theoretical and experimental foundation for the future fabrication of innovative implants.

A successful porous metal implant material improves bone function and stimulates bone tissue regeneration in damaged regions. Ideal bone implant features include the following (Hutmacher, 2000; Wang et al., 2016; Shaunak et al., 2017): 1) biocompatibility; 2) the capacity of cells to cling together, grow, and mature; 3) density and porosity in three-dimensional space that allows cell development and the movement of nutrients and waste products; and 4) mechanical properties that match those of tissues to eliminate stress shielding. Therefore, the key features of the design of porous metal implant materials include the correct analysis of porosity, pore size and pore connectivity. These structural features significantly affect the physical and biological characteristics of metal implant materials that are necessary to obtain satisfactory clinical results.

### Pore size

According to the previously published literature (Ai et al., 2021; Zou et al., 2021, 2022), it is still not clear how the pore size affects bone growth. Micropores are beneficial to the hypoxic environment, which leads to the development of osteoinduction before osteogenesis, while macropores with good vascularization lead to immediate osteogenesis (no previous cartilage formation) (Karageorgiou and Kaplan, 2005). According to research, the minimal pore size necessary to stimulate bone ingrowth is in the range of 100–150 μm (Deb et al., 2018; Abbasi et al., 2020), and the minimum value is determined by the size of osteoclasts responsible for bone resorption. Due to the narrow range of pore size, single cells can stretch and connect stomata, inhibit the inward invasion of cells, and limit the diffusion of wastes and nutrients into the cell network. Pores in the range of 200–400 μm in size are thought to promote osteoblast adhesion, migration, and proliferation. Implants with pores larger than 300 μm have been generally considered to dramatically enhance new bone and capillary formation (Ran et al., 2018), and better vascularization may promote osteogenesis.

Additionally, there was no significant increase in vascularization at pore diameters greater than 400 μm, which showed that 400 μm was the upper limit of vascularized pore size (Bai et al., 2010). Some studies have found that pores larger than 900 μm have limited cell bridging ability (Dziaduszewska and Zielinski, 2021). Smaller pores may have a more significant surface area that allows more bone tissue to form (Tan et al., 2017). Capillarity, vascularization, and cell-matrix interactions are supported by micropores with pore diameters less than 10 μm. The 150-900-μm pores allow waste and nutrients to diffuse into the cellular network (Deb et al., 2018). The ideal pore size has been shown to be in the range of 300–600 μm in studies of several elements of bone development, vascularization, mechanical strength, and permeability (Karageorgiou and Kaplan, 2005; Taniguchi et al., 2016; Tan et al., 2017).

Research has shown that when the pore diameters of titanium alloy implants manufactured using 3D printing technology are 400–1,000 μm, the implants have good osteogenesis ability. Fukuda et al. (2011) has used selective laser melting (SLM) to manufacture porous titanium implants with pores sizes in the range of 500–1200 μm. When the pore diameter was 500 μm, the area and length of bone formation demonstrated outstanding bone induction. Taniguchi et al. (2016) has shown that at 65% porosity, the osteogenesis performance of implants was better for pore diameters of 600 μm 300 and 900 μm. In contrast, the bone development was inferior for implants with 300 μm pore diameter. Wieding et al. (2015) reported that metatarsal restoration was successful when the pore diameter was 700 μm.

Similarly, Zhang et al. (2022) created porous implants with pore sizes of 400 μm, 700 μm, 900 μm, and 70% porosity using the SLM method, and the results revealed that pore sizes in the 600–700 μm range and porosities in the 70%–90% range offered promising outcomes for bone defect repair. Wang C. et al. (2021) used electron beam melting (EBM) printing technology to prepare implant materials with various pore sizes. They discovered that porous implants with designed pore diameters of 1,000 μm boosted stem cell adherence, growth, and osteogenesis, and this resulted in improved bone regeneration and vasculature. To summarize, pore size affects bone development dynamics and vascularization capacity in vascularized tissues such as bone. Small pores are required for bone formation to maximize available surface area and speed tissue regeneration. However, large pores enhance cell mobility, nutrition transfer and tissue oxidation (Cavo and Scaglione, 2016) as shown in Table 1.

**TABLE 1:** Porosity and pore size of 3D printed titanium scaffold for bone regeneration (pore size is expressed as range or average pore size).

Fabrication technique	Pore size range	Porosity	Type of study	Optimum pore size	References
SLM	500–1200 μm		*In vivo*	500 μm	Fukuda et al. (2011)
SLM	300 μm, 600 μm, 900 μm	65%	*In vivo*	600 μm	Taniguchi et al. (2016)
SLM	700 μm	orientated perpendicularly in all three spatial directions	*In vivo*	700 μm	Wieding et al. (2015)
SLM	400 μm, 700 μm, 900 μm	70%	*In vivo*	600–700 μm	Zhang et al. (2022)
				70%–90%	
EBM	800 μm, 900 μm, 1000 μm	86.30%–94.22%	*In vitro*	1000 μm	Wang et al. (2021a)

### Porosity and pore structure

The main factors that affect bone formation are the distributions of the size and shape of the pores and the porosity and internal structure of the bone (Chen H. et al., 2020; Wang et al., 2022). Numerous studies have been conducted on cellular response and osseointegration related to titanium implants with various pore diameters and porosities, and their optimal values remain a contentious topic (Zhang et al., 2022), possibly because results were inconsistent when researchers used implants with various porous structures and implanting them in different locations in the human body.

Porosity is defined as the ratio of the pore volume to the overall volume of material (Lei et al., 2020). Its value depends on the size and structure of the pores and their branched structure. The functions of porosity include regulating cell motility, oxygen, and nutrient delivery and offering a surface area for creating new tissue (Dziaduszewska and Zielinski, 2021). However, porous structure has contradictory effects. More porosity causes better bone ingrowth, but over time, it weakens the mechanical strength of the material. By controlling porosity and preferred pore size, the proper balance between mechanical characteristics and microstructure can be achieved to boost the effectiveness of implants *in vivo* (Kapat et al., 2017). In the laboratory, osteogenesis is accelerated by limiting cell growth and forcing cells to stick together. In contrast, increased porosity and pore size promote bone growth *in vivo* but at the expense of their mechanical properties, which sets a practical upper limit for pore size and porosity (Karageorgiou and Kaplan, 2005; Babaie and Bhaduri, 2018). Most porous metals used for bone implants have good mechanical strength and low porosity (up to 40%). However, low porosity does not guarantee proper implant permeability, which is needed for proper blood flow and mineralization of human bone tissue (Dabrowski et al., 2010). Recent experimental studies have demonstrated that implants with porosity close to that of natural trabeculae bone (70–90%) can promote cell survival and stimulate bone formation, whereas greater porosity (>60%) is advantageous to osteogenesis. As a result, for the design of porous implants, pore diameter >300 μm and porosity >60% are recommended (Zhang et al., 2022).

The overall design of the implant can be separated into homogeneous design, gradient design, and design based on topology optimization, all of which simulate the heterogeneous hierarchical structure of bone. As AM technology advances, the development of triply periodic minimal surfaces (TPMSs) and Voronoi implants is accelerating (Chen H. et al., 2020). The Voronoi implant has greater unpredictability, and it is comparable to cancellous bone efficiency. Owing to their uniform and slick surface, TPMS implants have good permeability. Both designs have the characteristics that are required for medical implants (Chen H. et al., 2020). Fousova et al. (2017) used selective laser melting to prepare specimens of three types of Ti6Al4V alloys with porosity gradients and comprehensively characterized their microstructure, porosity, biocompatibility, and tensile and compressive mechanical properties. The dense part of the gradient structure had high strength and good mechanical properties, which simulated the behavior of human bones. Li et al. (2015) produced porous titanium with porosity of 30%–70% and average pore diameter of 100–650 μm by the diffusion-linking method, and the prepared porous titanium with anisotropic structure and porosity of 60%–70% can be used for trabecular bone repair. Liu et al. (2020) printed Ti6Al4V scaffold with curved structure and strut structure by SLM, with porosity of 65% and pore diameter of 650 mm. Two groups of scaffolds were inserted into the femoral condyle of rabbits and assessed for their potential to integrate with bone. The outcomes showed that while the mechanical qualities of the curved construction were inferior to those of the strut structure, their osteogenesis was superior. Deng designed four kinds of porous titanium alloy scaffolds with different structures, 65% porosity and pore diameter of approximately 650 μm and implanted them into the distal femur of rabbits. The findings demonstrated that pore structure had a significant impact on bone development; diamond lattice unit (DIA) structure had the most favorable effects (Deng et al., 2021).

The morphology (pore size, porosity, and porous structure) and mechanical characteristics of bone, as well as the gradient characteristics of adsorbed cytokines, make it challenging to fabricate biomaterial implants that fulfill the needs of specific application sites (Karageorgiou and Kaplan, 2005).

## Micro-arc oxidation of titanium and alloy implant materials

Titanium alloy is a biologically inert substance, unlike calcium phosphate-coated implants, which cannot bond sufficiently with adjacent bones or stimulate bone growth and antibacterial ability. In clinical practice, there is a strong demand for implant surfaces that promote bone formation and faster bone binding, thus reducing the implantation-to-loading time and boosting implant success (Pellegrini et al., 2018; Llopis-Grimalt et al., 2020; Smirnov et al., 2020). Because the naturally occurring titanium oxide film is too thin to enhance the biocompatibility of titanium metal implants, the titanium oxide layer formed on the surface of titanium is critical for its biocompatibility. For Ti-based metal implants, several surface treatment techniques have been developed to increase their biological activity (Li and Zhao, 2009).

Different results, such as strong mechanical adhesion between bone and implant, enhanced osteoinduction and osteoconduction, better wear and corrosion resistance, improved bioactivity and biocompatibility, and accelerated healing time, can be accomplished through various changes in the surface (Sasikumar et al., 2019). Surface modification technologies can be divided into subtractive and additive techniques according to their method of surface modification. Subtraction technology includes sandblasting (Boyan et al., 1998), acid etching (Zhang et al., 2020), polishing (Wennerberg and Albrektsson, 2009), and microlaser deformation (Zheng et al., 2020), and addition technology includes physical deposition techniques such as plasma spray coating (Cao and Liu, 2013), anodic oxidation by chemical treatment (Macak et al., 2005), micro-arc oxidation (Cimenoglu et al., 2011; Zhang Y. et al., 2018; Chen et al., 2019; Cui et al., 2021; Guan et al., 2021), hydroxyapatite and calcium phosphate coatings (Surmenev et al., 2014), and sol-gel technologies (Zhang et al., 2011). In chemical surface modification, electrochemical, chemical or biochemical reactions may occur at the interface between the titanium implant surface and solution (Pesode and Barve, 2021).

MAO is a new electrochemical surface modification technology that can form porous, relatively rough and firmly attached oxide films on the surface of valve metals, such as titanium substrates (Zhao Z. et al., 2009). The porous outer layer of oxide (sometimes called the “technical layer”) is mainly composed of a low-temperature X-ray amorphous phase. Below the technical layer is an important “functional layer,” which is a denser oxide prepared by Walsh et al. (2013) higher temperature modification. There is a very thin, dense and complex oxide layer (“barrier layer”) between the functional layer and the metal surface. The microstructure of MAO coatings is often relatively complex, while Ti MAO usually contains both anatase and rutile. Amorphous material is often also present. Machining the most common titanium alloy (Ti6Al4V) produces a large amount of mixed oxide aluminum titanate. Notably, in general, it is difficult to heat treat MAO coatings to change these structures because the required temperature range is usually higher than or at least close to the melting point of the metal substrate (Clyne and Troughton, 2018).

**FIGURE 2:**
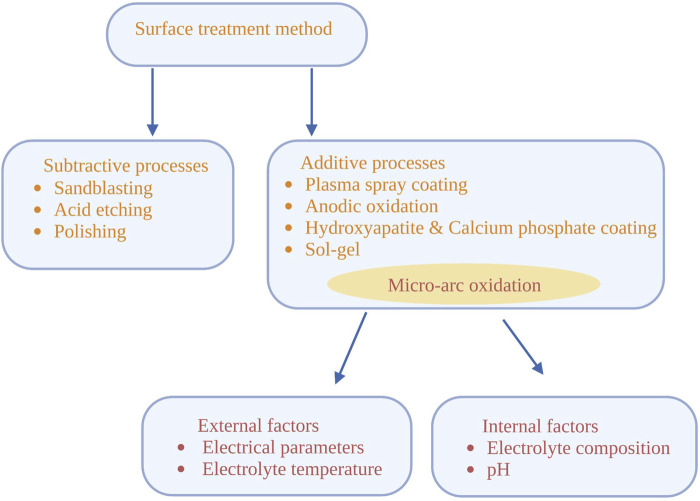
Surface treatment methods of titanium substrate and factors affecting micro-arc oxidation.

External factors (electrical parameters and electrolyte temperature) and internal factors (electrolyte composition and pH value) affect the creation and microstructure of the MAO film. In particular, it is very important to use appropriate design, concentration and electrochemical parameters of the electrolyte to obtain the specific phase composition and microstructure required by the coating (Figure 2).

## Electrolyte

The electrolyte is the most important of these MAO components because the plasma arc may be controlled by electrochemical processes connected to ionized chemical elements, which in turn affects the characteristics of the titanium oxide surface (Jung et al., 2014). The composition of the electrolyte is the most important factor that determines the microstructure and qualities of the MAO protective layer. Based on the probable reaction in the MAO process, there are two potential mechanisms for MAO coating development. Ions from one electrode migrate from the substrate/coating contact to the electrolyte while ions from the other electrode migrate inward, causing one electrode to grow outward and the other to grow inward (Wang et al., 2020). In recent years, domestic and foreign scholars have conducted extensive research on this topic (Table 2). Ti-based MAO electrolytes are mainly composed of acidic and alkaline solutions. However, due to the disadvantages of an unfavorable environment and difficult waste liquid treatment, alkaline solutions are gradually replacing acidic solutions. The standard solution includes a silicate electrolyte system, a phosphate electrolyte system, an aluminate electrolyte system and a sodium tetraborate electrolyte system. In recent years, organic phytate has been selected as the electrolyte for MAO of Ti-based alloys.

**TABLE 2:** MAO treatment of titanium and its alloys with different electrolyte compositions.

Different electrolyte systems	Electrolyte composition	Substrate	Surface morphology	Results of XRD	Outcome	References
Silicate electrolyte system	Na_2_ (EDTA), CaO and Ca(H_2_PO_4_)_2_, H_2_O	Pure titanium	Porous microstructure, the pore size is around 1–5 μm	Anatase and rutile	Grows fast and corrodes fast in SBF solution	Zhang et al. (2008)
	Na_2_SiO_3_·9H_2_O, (NaPO_3_)_6_, NaAlO_2_	Ti6Al4V discs	Nano-scale TiO_2_ grains, of different size, ranging from several nm to tens nm	Rutile and a small amount of anatase TiO_2_	The adhesion strength of coating interface is found to be about 70 MPa	Wang et al. (2006)
	Sodium silicate (Na_2_SiO_3_·9H_2_O) and calcium glycerol phosphate (C_3_H_7_CaO_6_P)	Ti6Al4V alloys	Calcium phosphate electrolyte produces a thicker, more compact MAO layer than silicate	The silicate electrolyte consists of TiO_2,_SiO_2_, Ti_3_(PO_4_)_4_, TiP_2_O_7_, and the calcium phosphate electrolyte comprisingTiO_2_, CaO, CaTiO_3_, Ti_3_(PO_4_)_4_, TiP_2_O_7_ and Ca_2_P_2_O_7_	The CaP apatites can integrate with human bone tissue and promote bone growth	Wang et al. (2020)
Phosphate electrolyte system	(NaPO_3_)_6_–NaF–NaAlO_2_	Ti6Al4V alloy	As treatment duration increases, coating development slows and roughens	Anatase, rutile and AlPO_4_ phases	The adhesion strength of substrate/coating interface is about 40 MPa	Wang et al. (2004)
	β-glycerophosphate disodium salt pentahydrate and calcium acetate monohydrate	Pure titanium plates	Macro-porous, Ca- and P-containing titania-based films were formed on the titanium substrates	Rutile and anatase	Ca- and P-containing, micro-arc oxidized titanium implants have the capability to induce bone-like apatite	Song et al. (2004)
	CaCl_2_, KH_2_PO_4_	Pure Ti	MAO micro-arcs decrease when CaCl_2_ concentration increases, while nanocrystals grow	XRD patterns didn’t show anatase or rutile titania (TiO2) production	First, a single MAO coating procedure was proposed to generate crystalline HAP coatings on Ti substrates	Kim et al. (2007)
	Citric acid, ethylene diamine, and ammonium phosphate	Ti6Al4V alloy	An HA crystalline peak could not be detected by XRD	Coated with TiO_2_ film and hydroxyapatite	Improved bioactivity, cell adhesion, and viability while retaining film-substrate bonding	Hong et al. (2011)
	H_2_SO_4_-H_3_PO_4_	Pure titanium and Ti6Al4V	Ti6Al4V has a cortical morphology with irregular worm-like slots, unlike MAO/Ti		MAO films were successfully produced on pure Ti and Ti6Al4V materials at 180 V. MAO substantially improved the corrosion resistance of untreated materials	Fazel et al. (2015)
	Na_3_PO_4_ and K_3_PO_4_	Pure titanium	K_3_PO_4_ electrolyte’s oxide layer was rougher than Na_3_PO_4_’s	Anatase and rutile crystalline phases	Attachment and multiplication of osteoblast cells to K_3_PO_4_’s oxide layer were better than in Na_3_PO_4_	Jung et al. (2014)
Aluminate electrolyte system	Aluminate solution	Ti6Al4V alloy	After MAO treatment, Ti6Al4V substrate microstructure is unaltered and no hardening zone is identified	TiO_2_ rutile and TiAl_2_O_5_ compounds	Nanohardness and elastic modulus rise from coating surface to inside	Wenbin et al. (2002)
	NaAlO_2_ electrolyte	Pure titanium	Increasing NaAlO_2_ lowers micropores, increases the quantity and size of sintered disks, and roughens the surface	Mainly composed of TiO_2_, rutile and anatase	The surface of the coating is rough, and the corrosion rate first decreases and then increases	Ping et al. (2016)
Sodium tetraborate electrolyte system	Na_2_B_4_O_7_·10H_2_O	Pure titanium slices	Cortex-like layers with pores and slots	Mostly rutile	Cortex-like coatings with interior pores and slots are more wettable than volcanic coatings	Liu et al. (2013)
	Li_2_B_4_O_7,_Na_2_B_4_O_7_ and K_2_B_4_O_7_	Pure titanium disks	Novel “cortex-like” micro/nano dual-scale structured TiO_2_ coating	Rutile with a little anatase	Promotes stem cell adhesion, spreading, and differentiation, and leads to excellent osseointegration	Li et al. (2018)
Phytic acid	Phytic acid, KOH, EDTA-Na_2_, Ca(CH_3_COO)_2_	Ti6Al4V plates	Typically porous structure	Anatase- TiO_2_,rutile-TiO_2_ and perovskite-CaTiO_3_ phases	Porous TiO_2_ ceramic layer containing calcium and phosphate was prepared by MAO on Ti6Al4V alloy	Qiao et al. (2016)
	EDTA-ZnNa_2_, KOH, and phytic acid	Ti6Al4V plates	Typical porous structure	Anatase and rutile	MAO coating combines Zn and P, and phytic acid concentration impacts Zn and P content, which is beneficial	Wang et al. (2018b)
	NaOH and Na_12_Phy	Ti6Al4V	Typical porous structure and the pore size is about 3 μm in diameter	Anatase TiO_2_	MTT tests showed good biocompatibility	Zhang et al. (2015)
	Phytic acid	Ti6Al4V alloys	Porous structure with tiny micropores and great hydrophilicity	Rutile, anatase, TiP_2_O_7_ as well as some OH- groups	MC3T3-E1 Pre-osteoblasts had excellent cytocompatibility in viability, adhesion, proliferation and differentiation	Wang et al. (2017)

### Silicate electrolyte system

In the process of MAO, the adsorption capacity of various anions is different. In the standard electrolyte system, silicate ions can quickly form a passivation film on the substrate surface, which makes the MAO reaction easier and the prepared coating thicker while allowing greater surface roughness and lower adhesion. MAO films made of titanium alloy in silicate electrolyte systems mainly contain anatase, rutile and amorphous silica. In an electrolyte containing Ca^2+^, H_2_PO_4_ and SiO^2+^, silicon-doped bioactive ceramic coating was formed on the surface of pure titanium using the MAO method (Zhang et al., 2008). Compared with the standard MAO film, the MAO film containing silicon had almost the same porous structure and phase composition. Wang et al. (2006) used a bipolar pulse power supply to form a bioceramic coating on the surface of Ti6Al4V alloy by MAO in a system solution that included Na_2_SiO_3_. The layer was mainly composed of rutile and a small amount of anatase, and it was doped with amorphous compounds of electrolyte components. A bioceramic film was formed on the surface of Ti6Al4V alloy in silicate electrolyte and calcium phosphate electrolyte by MAO technology (Wang et al., 2020). Research was performed to determine how the content of the solution and the positive voltage affected the microstructure of the coating as well as its resistance to corrosion. According to the findings, the MAO film that was prepared using silicate electrolyte expanded quickly and corroded in SBF solution. However, the MAO film that was prepared using calcium phosphate electrolyte had good surface characteristics and resisted corrosion. It is an ideal candidate material for applications in bone implants and dental replacement materials.

### Phosphate electrolyte system

Phosphate-based electrolytes provide smoother surfaces and thinner coatings than those of silicate electrolyte systems, and they have excellent adhesion. The MAO film formed in phosphate electrolyte system based on titanium and its alloy is primarily made up of rutile and anatase. It is dense, wear-resistant and corrosion-resistant.

Wang et al. (2004) produced a ceramic coating on the surface of Ti6Al4V alloy in (NaPO_3_)_6_–NaF–NaAlO_2_ solution by using constant current MAO technology. As the treatment time increased, the coating grew quickly at first and then slowly, its appearance became rougher over time, the phase transition from anatase to rutile occurred. The interface between the substrate and the coating had a bonding strength of approximately 40 MPa. This was based on both the bonding force of the coating to the substrate and the cohesive strength inside the coating. Under various applied voltages (200–500 V), Song et al. (2004) used MAO to form titanium oxide coatings containing Ca and P on commercial titanium substrates in electrolyte containing β-glycerophosphate pentahydrate and calcium acetate monohydrate (CA). A macroporous titanium dioxide base film containing Ca and P formed on the titanium substrate. Micro-arc titanium oxide implants containing Ca and P have the ability to induce bone-like apatite (biological activity) in SBF. The 1.5 SBF decreased the apatite induction time, and apatite was generated on the surface of 350 V oxide films, which demonstrated that Ca and P play a similar role in titanium films and SBF. A more concentrated SBF was utilized to reduce the apatite induction time and examine the impact of the apatite formation ability on the oxide layer properties.

Kim et al. (2007) was the first to prepare nanocrystalline hydroxyapatite (HAp) films on titanium surfaces by one-step MAO using an electrolyte containing Ca^2+^ and P^5+^ ions. At high density, hydroxyl groups can combine with Ca^2+^ and PO_3_ to form crystalline HAP. Because of the strong crystallinity of hydroxyapatite film, the material has strong biocompatibility and good potential in orthopedic and dental restoration applications. Hong et al. (2011) conducted MAO experiments using Ti6Al4V alloy as the substrate, different concentrations of citric acid, ethylenediamine, and ammonium phosphate as electrolytes, and various voltages. The results revealed that the films containing hydroxyapatite were biologically active, the adhesive force with the Ti substrate was comparable to that of commercial products and adhesion was unaffected by TiO_2_ film thickness and morphology. The adhesion and proliferation of MC3T3-E1 osteoblast cells were regulated by Ca and P ion concentrations and the electrolyte fraction.

MAO was used by Fazel et al. (2015) to process pure Ti and Ti6Al4V samples at 180 V in an electrolyte of H_2_SO_4_-H_3_PO_4_. A cortical morphology with irregular worm-like slits was observed on seen, in contrast to the volcanic morphology of the oxide layer on pure Ti. The corrosion resistance of untreated samples was dramatically increased by the MAO procedure.

To demonstrate that the rough and porous surface of the titanium oxide film generated by MAO coating is critical for cell attachment, Jung et al. (2014) formed titanium oxide layers on titanium samples using two different electrolytes comprising Na_3_PO_4_ and K_3_PO_4_. Because of the high fraction of micropores, the surface of the oxide layer generated in the K_3_PO_4_ electrolyte was rougher than that formed by the Na_3_PO_4_ electrolyte. Osteoblast adhesion and proliferation to the oxide layer generated in the K_3_PO_4_ electrolyte were superior to that in the Na_3_PO_4_ electrolyte.

### Aluminate electrolyte system

MAO can introduce alumina into the membrane as aluminate electrolyte solution. The MAO film generated in aluminate electrolyte solution offers excellent wear resistance because aluminum oxide typically has incredible hardness and stability. A thick ceramic oxide film was prepared on the surface of Ti6Al4V by MAO in aluminate solution. The residual matrix structure of the Ti6Al4V alloy remained unchanged, and no hardening zone was found in the matrix. In addition, oxygen atoms did not migrate into the alloy matrix, and the oxide film was mainly composed of TiO_2_ rutile and TiAl_2_O_5_ compounds (Wenbin et al., 2002). The MAO method was used to deposit a layer of titanium dioxide on pure titanium substrate in NaAlO_2_ electrolyte (Ping et al., 2016). The concentration of sodium aluminate had a direct impact on the performance of the MAO process and the quality of the film. By increasing the content of NaAlO_2_, the growth rate of the MAO film on the pure titanium surface was significantly increased and promoted by the working voltage. Rutile and anatase TiO_2_ constitute the majority of the MAO coating. With increasing NaAlO_2_ concentration, the TiO_2_ phase content decreases. The number of micropores on the coating surface decreases, and the surface of the layer is rough. The corrosion rate first decreases and then increases. However, the application of aluminate-based electrolytes is restricted due to their poor stability and ease of hydrolysis.

### Sodium tetraborate electrolyte system

Studies have shown that cells tend to grow inward on porous and rough surfaces, and porous structures help to maintain the connection between implants and cells. Few investigations have studied alternative forms of MAO modification; previous studies have mostly focused on volcanic coatings on titanium substrates. By using sodium tetraborate as electrolyte in the MAO process, a titanium dioxide coating with cortical grooves was formed on a titanium substrate (Liu et al., 2013). Cortical coatings with internally communicating pores and gaps have better wettability than conventional volcanic coatings. Cell culture experiments showed that both cortical-like and volcanic coatings exhibited good cell adhesion, which indicated that they had good biocompatibility. The spreading of cells on the cortical coating was superior to that in the control group. The cell growth of the cortical coating followed cell ingrowth into the deep groove, which was beneficial to cell retention and implant stability.

Micro/nanostructures can promote the adhesion of proteins and cells, regulate angiogenesis growth factors, and affect the proliferation, differentiation and gene expression of osteoblasts and osteoblast-like cells (Raines et al., 2010). In addition, the three-dimensional porous structure helps to provide nutrition and support waste excretion. Li et al. (2018) adopted the tetraborate MAO method to prepare a novel “cortex-like” micro/nano double-scale TiO_2_ coating on the surface of titanium. This structure combined microslots with nanopores, and it showed super hydrophilicity. *In vitro* investigations revealed that the “cortex-like” form promoted bone marrow mesenchymal stem cell attachment, migration, and maturation and increased matrix mineralization. *In vivo* animal tests revealed that this “cortical” layer improved bone integration. Excellent cellular compatibility and bone attachment were achieved thanks to the hydrophilic synergy of the dual-scale structure and the “cortical” titanium dioxide covering, which may contribute to the success of implants.

### Phytic acid

Experts have recently discovered that environmentally beneficial organic phytate can be utilized as electrolyte for titanium alloy MAO. Phytic acid is a nontoxic organic macromolecule found in beans, grains, oil seeds, nuts, spores, tubers, pollen, and organic soil (Zhang et al., 2015). Phytic acid and sodium phytate have many health benefits and can be employed as anticancer agents, dietary additives, and chelating agents (Dost and Tokul, 2006). An orthogonal test can quickly and accurately determine the influence of process factors on target parameters. We can determine the order of the influence of many technical aspects, from the most important to the least important. Orthogonal experiments are needed to determine how cations and anions enter the MAO layer (Wang et al., 2017). In solutions with phytic acid concentration of 15 g/L and KOH concentration of 10 g/L, the effects of EDTA-Na_2_ concentration, Ca(CH_3_COO)_2_ concentration, current density and treatment time on the content of calcium and phosphorus in the Ti6Al4V alloy micro-arc oxidation anode film were studied by orthogonal experiments with four factors and three levels (Qiao et al., 2016). The results showed that diffusion and electromigration processes allowed calcium to enter, and the amount of phosphorus in the anode coating was strongly dependent on the amount of Ca(CH_3_COO)_2_. In the electrolyte containing EDTA-ZnNa_2_, KOH and phytic acid, the orthogonal experiment with four components and three levels was adopted to explore the influence of various technological factors on the amounts of Zn and P (Wang Y. et al., 2018). The results showed that Zn entered the MAO membrane with P from phytic acid, and P mainly participated in membrane formation through diffusion. The concentration of phytic acid was the key factor that determined the contents of zinc and phosphorus in the film, which contributed to the formation of the film.

Zhang et al. (2015) used sodium phytate as an electrolyte to apply MAO and successfully formed anatase TiO_2_-based anodic oxidation coating on the surface of Ti6Al4V alloy. Sodium phytate contributed to the formation of the coating, and phytate was formed in the anode coating. The MTT test showed that both Ti6Al4V and Ti6Al4V treated with MAO were biocompatible. Wang et al. (2017) used phytic acid as a unique titanium electrolyte. The results showed that the MAO film was composed of rutile, anatase and Ti_2_O_7_ and has good hydrophilicity and micropore structure. Compared with the untreated group, osteoblasts treated with MAO showed good cell compatibility in activity, adhesion, proliferation and differentiation, which may have been related to surface morphology and phase structure. All these results further verified that Ti6Al4V alloy treated by MAO in phytic acid solution can be used in medical implants.

## Electrical characteristics and power mode

When MAO is performed, the morphology and structural characteristics of the anode film are affected by the electrolyte composition and process electrical parameters, such as the treatment time, current density, frequency, temperature of the electrolyte and applied voltage (Pesode and Barve, 2021). However, there is a correlation between MAO process parameters that makes it more difficult to change them.

Huang et al. (2007) produced Ca-P titanium dioxide with rough porous structure by applying MAO technology at various voltages. The results demonstrated that the pore size increased with increasing applied voltage; samples prepared at 240 and 350 V exhibited greater bonding strength than those prepared at higher voltages. The modulus of elasticity and residual stress increased as the applied voltage increased. Typical compositions of the layer were anatase and rutile, but at higher voltages of 400 and 450 V, a new CaTiO_3_ phase formed. The total elastic modulus of the porous layer was significantly less than that of pure titanium, which reduced the stress shielding effect. In summary, MAO can dramatically enhance the mechanical qualities of titanium implants, which is advantageous for its widespread application.

Under various application voltages (250–500 V), Song et al. (2004) found that MAO produced a macroporous titanium oxide-based coating containing Ca and P on commercial titanium substrates. The *in vitro* bioactivity of oxidized materials was evaluated by immersing them in SBF with varying quantities of simulated body fluids and measuring their capacity to induce apatite formation. The experiment showed that the phase, Ca, and P content, shape, and thickness of the films were strongly related to the applied voltage. At low voltage, the oxide layer consisted primarily of anatase (250 V). With increasing applied voltage, the rutile phase appeared gradually, and the oxide layer became a mixture of anatase and rutile. X-ray diffraction (XRD) did not detect the phase containing Ca and P below 350 V. Experiments *in vitro* indicated that micro-arc titanium oxide implant materials containing Ca and P could create bone-like apatite (bioactive) in SBF.

By changing the voltage and time settings, Montazeri et al. (2011) adapted the MAO technique to an electrolyte containing Ca and P to create HA coatings directly on Ti6Al4V. The duration of voltage treatment played an important role in the formation of the HA phase, and minimum voltage was needed to form HA with titanium dioxide. Prolonging the operation time produced more HAs.

Electrical parameters such as AC or DC can be used in the MAO process for standard power modes. The coating process was more effective when AC power was used rather than DC power. Typically, pulsed bipolar or pulsed monopolar power sources are utilized for MAO treatment of titanium and its alloys. Currently, pulsed bipolar MAO power supplies are used extensively due to the increased thickness of the coatings and their excellent adherence to titanium substrates (Pesode and Barve, 2021). Current density is one of the parameters that has the greatest influence on coating performance and microstructure. With the increase in density, the thickness of the film increased, the microstructure became more accurate, and the surface roughness decreased (Yangi and Wu, 2012; Clyne and Troughton, 2018). Previous research demonstrated that frequency affected the performance of MAO coatings. At lower frequencies, the coatings were rougher with larger pores, and AC conditions were more effective than DC conditions for coating formation. As the duty cycle grew, the discharge period within a single pulse increased, the thickness of the film layer increased, more holes were produced, and the roughness increased when the voltage and frequency were constant (Torres-Ceron et al., 2019).

## Combined application of MAO and other treatment methods

**FIGURE 3:**
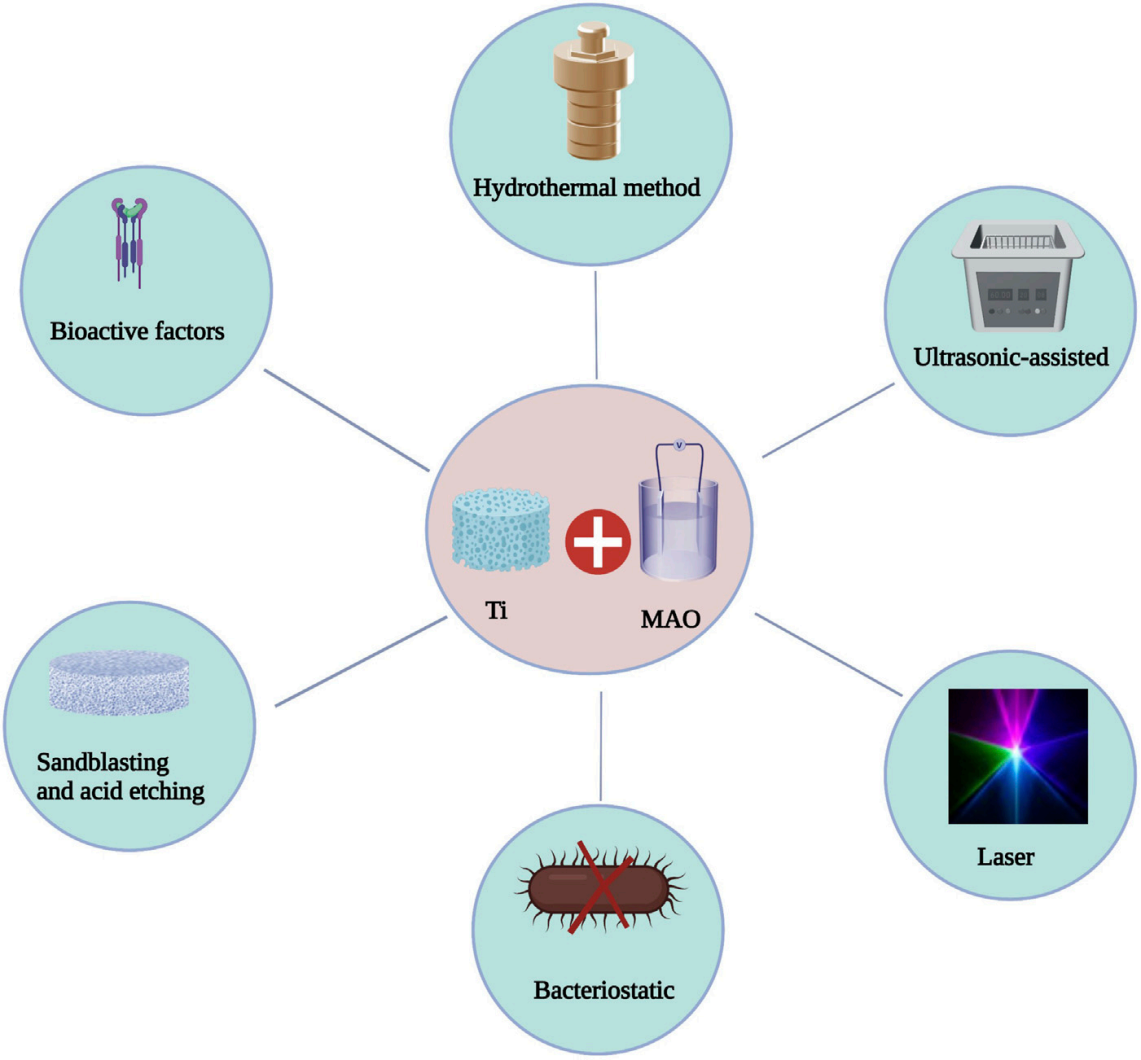
Combined application of MAO and other treatment methods.

By modifying the process parameters (voltage, current, electrolyte concentration, and treatment time) in the MAO process, the thickness, microstructure, roughness, and concentration of bioactive atom in the MAO layer may be easily manipulated. The chemical properties of the MAO surface can also be employed to further enhance the bioactivity of MAO-treated materials by combining MAO with other modification approaches (Deng et al., 2010) (Figure 3). The theory of MAO surface interactions with other modification techniques is not yet understood (Table 3).

**TABLE 3:** Combined application of MAO and other treatment methods.

MAO combined with other treatments	Substrate	Surface morphology	Outcome	References
Combined application of MAO and hydrothermal method	3D-printed Ti6Al4V scaffolding	Micro-nano hybrid coating with moderate roughness	Enhance biocompatibility, osteogenesis, and osseointegration	Huang et al. (2021)
	3D Printed Macroporous Ti6Al4V Implants	Nanofibers on microporous walls	Improve three-dimensional porous Ti64 scaffold apatite *in vitro* and osseointegration *in vivo*	Xiu et al. (2017)
Combined application of MAO and ultrasound	Ti6Al4V alloy	Homogenized coating structure	Improve corrosion and wear resistance of coating	Xu et al. (2021)
Combined application of MAO and laser	Ti6Al4V titanium alloy plate	Microgrooves reduce liquid-solid contact angle and boost surface roughness	Significantly increase the proliferation and differentiation of MC3T3-E1 cells	Zheng et al. (2020)
	Ti6Al4V alloy	Pores are uniformly distributed, tiny, and thick	Higher hardness and better wear resistance	Wu et al. (2020)
MAO and bacteriostatic treatment	Grade 4 quality Cp-Ti discs	Adding calcium, phosphorus, and silver ions	Improve antibacterial efficiency while maintaining biological activity	Teker et al. (2015)
	Ti6Al4V titanium discs	Hydroxyapatite (HA) and Ag+	Good antibacterial activity	Muhaffel et al. (2016)
	Commercially pure titanium	Micro-porous with pore diameters of 1–4 μm	Reduce planktonic bacteria and *Staphylococcus aureus* in culture	Zhang et al. (2016)
	Ti6Al4V	Surface becomes smoother as pores get smaller and more average	Ti-MAO-Cu_2_O group has the strongest antibacterial ability	Zhao et al. (2016)
	Ti6Al4V plate	Porous, uneven microstructure	Reduced planktonic and bacterial adherence	Zhou et al. (2019)
	Commercial Ti6Al4V plates	Double-layer structure, outer amorphous, inner polycrystalline	Good antibacterial activity is related to its strong electronic storage capacity	Wang et al. (2021c)
Combined application of MAO with sand blasting and acid etching	Titanium discs	Irregular valleys, micropores, and roughness	Enhanced biocompatibility, favourable for osteoblast differentiation	Deng et al. (2010)
MAO combined with other bioactive factors	3D-printed 600 μm pore Ti6Al4V plate	A numerous homogenously distributed pores	Promote osteogenesis and angiogenesis	Teng et al. (2019)

### Combined application of MAO and hydrothermal methods

The hydrothermal method is an effective method for inorganic synthesis and material treatment. It uses an aqueous solution as the reaction system in a specifically designed closed reactor (autoclave) and applies heat and pressure to dissolve and recrystallize normally insoluble materials. MAO can produce porous and firmly adhered titanium oxide coatings on titanium implant materials, thus enhancing the adhesion of titanium implant materials to bone. After hydrothermal treatment, the precipitation of HA on the titanium oxide film containing Ca and P ions produced a bioactive surface, so titanium oxide showed good bone adhesion and pushing force. After hydrothermal treatment, enough hydroxyl groups can be formed on the surface of the sample to serve as nucleation points of hydroxyapatite crystals. Additionally, Ca and P ions combine with hydroxyl groups, which can be converted into hydroxyapatite crystals under the applied temperature and pressure.

Huang et al. (2021) prepared multifunctional micro/nano hybrid coating on the surface of 3D printed porous Ti64 by MAO and a hydrothermal method (HT) and studied the surface morphology, chemical composition and surface/cell interaction of the obtained coating. The surface roughness of the modified sample was moderate, the biocompatibility was improved, and the osteogenic ability was enhanced. Compared with hydrothermal surface treatment, MAO surface treatment had better biocompatibility and osteogenic ability. *In vitro* experiments showed that all Ti64 implants modified by mixed coating could enhance the adsorption of protein and the activity, adhesion and differentiation of MC3T3 osteoblasts. *In vivo* experiments showed that the mixed coating could promote early osseointegration. In contrast, Ti64 treated by MAO had the best biological activity and the strongest osseointegration ability. This had important theoretical significance and provided an opportunity potential applications for improving the bioactivity of titanium implants.

MAO-HT treatment was initially applied to a porous metal scaffold that was formed using 3D printing by Xiu et al. (2017). In addition, they developed a cylindrical Ti6Al4V implant with 640 micropores and 73% porosity using the electron beam melting technique. Following MAO-HT treatment, porous Ti6Al4V implants showed multiscale micro/nano morphology and a large amount of CaP. Compared to the untreated implant, the treated implant produced more apatite. The two types of implants had different patterns of bone growth: the treated implant had a pattern of contact osteogenesis, and the bone grew on its surface; the untreated implant formed bone at its distal end. Following MAO-HT treatment, the three-dimensional porous Ti6Al4V implant exhibited biological activity, which considerably enhanced its ability for apatite induction *in vitro* and bone integration *in vivo*.

### Combined application of MAO and ultrasonic assistance

MAO is widely used to build wear-resistant and corrosive environment films on titanium and titanium alloys; however, heterogeneity is inevitable. Xu et al. (2021) treated Ti6Al4V by ultrasonic-assisted micro-arc oxidation and compared it with Ti6Al4V by micro-arc oxidation without ultrasonication. They thoroughly studied the effects of different ultrasonic treatment times on the microstructure and characteristics of the coating. The results showed that ultrasonic-assisted MAO of Ti6Al4V could homogenize the coating structure and improve the corrosion resistance and wear resistance of the coating in the human body.

There are limited investigations on the biomedical application of ultrasonic-assisted MAO of Ti6Al4V alloy, and the influence of ultrasonic assistance on the morphological features of the oxide layer on Ti6Al4V alloy is still unknown. In addition, in light of the excellent properties of MAO films prepared on Al and Mg alloys by ultrasonic-assisted methods and the extensive use of Ti alloys in the biomedical field, further research into the characteristics of MAO films prepared on Ti6Al4V by ultrasonic-assisted methods is essential (Xu et al., 2021).

### Combined application of MAO and lasers

Traditional surface treatment technology has some disadvantages, including low efficiency, high cost and difficult processing methods. Laser machining is an economical, efficient and rapid method to create and adjust complex topography to enhance wettability. For many years, scientists have paid more attention to the surface roughness and chemical surface treatment of material, and given less attention to the biological consequences and surface wettability. To promote long-term physical benefits, the optimal contact angle range for cell attachment is in the range of 40–60° (Lee et al., 1998; Faucheux et al., 2004; Zheng et al., 2020). Zheng et al. (2020) studied the wettability and surface morphology of the Ti6Al4V surface by laser processing microgrooves to observe the adhesion, proliferation, differentiation and cell morphology of mouse osteoblast MC3T3-E1 cells and determine whether the laser-treated surface could improve the biocompatibility of cells. The results demonstrated that the surface with laser microgrooves decreased the liquid‒solid contact angle and increased the surface roughness. The osteoblasts proliferated and differentiated sufficiently, most cells grew along the microgrooves, and the improved wettability provided a favorable environment for cell growth. Microgrooves play a vital function in cell contact guidance, and their characteristics regulate cell activity; therefore, they should be thoroughly researched.

Laser surface melting (LSM) is a new surface treatment method that may induce a nonequilibrium phase on the titanium alloy surface due to its rapid cooling rate. With LSM, the host material remains unchanged, and only the surface microstructure changes. Wu et al. (2020) prepared porous TiO_2_ ceramic coatings with high silicon and phosphorus contents on the surface of Ti6Al4V alloy by LSM and MAO. They studied the effect of LSM pretreatment on the morphology, composition and mechanical properties of MAO films. The results showed that the LSM-MAO coatings had more uniform pore distributions and smaller pores and were thicker than MAO coatings. The LSM-MAO coatings were harder and had better wear resistance than the MAO coatings. LSM is considered an appropriate and effective pretreatment method to obtain high-quality MAO coatings on titanium alloys.

### MAO and bacteriostatic treatment

Although the application of biocompatible coatings such as HA to accelerate bone healing and improve bone integration with metal implants is challenging, infection related to implants is still a significant risk. Thus, inhibiting bacterial colonization on the surface of implants has become one of the primary concerns of their successful use Li and Zhao (2009). The biocompatibility, wear durability, and corrosion resistance of MAO films manufactured using a certain electrolyte are all outstanding. Silver (Ag), zinc (Zn), and copper (Cu) are the most often utilized inorganic antibacterial additions in MAO process electrolytes (Zhao L. et al., 2009; Ning et al., 2015). Since silver ions (Ag^+^) react with protein thiol groups and adhere to bacterial DNA, they have excellent antibacterial powers against many bacteria (Simchi et al., 2011). Therefore, the application of Ag coatings on the surface of implant materials has become an interesting biomedical business strategy. The Ag^+^ concentration in the antimicrobial coating of an implant must be strictly managed because of the potential for cytotoxicity at high Ag^+^ concentrations and poor antibacterial activity at low values (Antoci et al., 2008; Teker et al., 2015). MAO was performed at 380 V in electrolyte containing Ca, P, and Ag ions, and adding Ag to multilayer coatings (biocompatible compound layer and underlying TiO_2_ layer) improved antibacterial efficacy while maintaining biological activity (Teker et al., 2015). Multilayer biomedical coatings were prepared on the surface of Ti6Al4V alloy by MAO under positive and negative voltages of 400 and 80 V (Muhaffel et al., 2016). Silver nitrate was added to the basic electrolyte containing calcium acetate hydrate and anhydrous disodium hydrogen phosphate, which improved the biological and antibacterial activity of the synthesized coating and caused silver nanoparticles to precipitate on the hydroxyapatite layer. The antibacterial efficiency of the silver-containing coating was more than four times that of coating without silver. Adding 0.1 g/L AgNO_3_ to the basic electrolyte is promising for the MAO process of titanium-based implants. Zhang et al. (2016) created an antibacterial TiO_2_ MAO coating doped with both zinc and silver by combining silver nanoparticles with zinc acetate. Compared with the control group composed of polished titanium, the coating significantly reduced the number of planktonic bacteria in the culture solution and inhibited the adhesion of *Staphylococcus aureus*. Nano Cu_2_O and nano ZnO have excellent antibacterial ability. Zhao et al. (2016) developed MAO coatings containing nanoparticles by introducing nano-Cu_2_O and nano-ZnO into the electrolyte. The antibacterial properties of the membrane were determined by exposing the sample to *Escherichia coli* and comparing Ti after treatment with MAO with untreated Ti. The results showed that the antibacterial activity of the Ti-MAO-Cu_2_O group was the highest of those in the study. These results imply that the antibacterial properties of titanium can be enhanced by incorporating nanoscale metal oxides into the MAO-coated film via an electrolyte. The antibacterial mechanism of inorganic antimicrobial agents is widely assumed to involve the release of antimicrobial ions to destroy bacteria; however, the continuous release of bactericidal ions often results in severe cytotoxicity and environmental concerns. To address these disadvantages of current materials, new effective, safe, environmentally benign and economical antibacterial materials must be developed soon (Wang R. et al., 2021).

Sodium tungstate has been utilized to resolve bacteria-related issues. Adding sodium tungstate to MAO coatings can be an effective method for achieving antibacterial effects. MAO coatings containing tungsten was used in the antibacterial field to confirm that a sodium tungstate-based MAO coating had various antimicrobial qualities. Zhou et al. (2019) used an alkaline electrolyte to synthesize an antibacterial MAO coating and examined the effect of sodium tungstate in the MAO coating on Ti6Al4V. For the first time, the possibility of producing an antibacterial MAO coating that contained tungsten by doping the electrolyte with Na_2_WO_4_ was established, and the antibacterial feature of the layer were discovered to be primarily dependent on sodium tungstate. The tungsten-containing MAO coating effectively hindered bacterial attachment, reduced the number of planktonic bacteria in the culture media, and hinted at the antibacterial action of the coating. To explore the antibacterial mechanism of tungsten-doped TiO_2_ coatings, Wang R. et al. (2021) used MAO technology to coat the surface of Ti6Al4V with tungsten-doped TiO_2_ with antibacterial properties. The microstructure of tungsten-doped and undoped TiO_2_ coatings was studied and compared. The antibacterial behavior of the tungsten-doped TiO_2_ coating was closely related to its amorphous surface. The MAO coating had a double-layer microstructure with an amorphous outer layer and a polycrystalline inner layer. The new electrolyte system containing tungsten was beneficial to the formation of amorphous layer with high electron storage capacity. The high electron storage capacity of the tungsten-doped TiO_2_ coating is related to its excellent antibacterial activity, which provides a new method for designing coatings with controllable antibacterial performance.

### Combined application of MAO with sandblasting and acid etching

Sandblasting plus acid etching has become the standard technique for the surface treatment of titanium implants, and it is a traditional modification technique used to enhance biological activity. Sandblasting, etching, or the combination of both can lead to the formation of various morphological features on the surface of titanium implants, including micron-scale changes such as dimples and ultra micron-scale changes such as waves or valleys. The surface roughness of implants is one of the factors that can successfully improve cell reactions *in vitro* and bone integration *in vivo*. The combination of micron-level and submicron-level roughness helps to increase osteoblast reactions, which may be partly due to the influence of surface protein-material interactions on the reaction of downstream cells (Cheng et al., 2014). However, sandblasting acid and etching change only the structure of the surface and have little effect on the chemical properties of the surface (Li et al., 2018). Deng et al. (2010) found that compared with MAO, MAO-HT and smooth surfaces, adherent osteoblasts showed stronger alkaline phosphatase (ALP) activity and osteocalcin (OC) production on MAO-SA surfaces. Experiments show that sandblasting and MAO can improve the surface morphology of titanium, increase the surface roughness, and make it a good place for cell growth and bone adhesion.

### MAO combined with other bioactive factors

Bone morphogenetic proteins (BMPs) can induce new bone formation, and the effectiveness of BMPs in osteogenesis depends on the control of carrier type and release rate. Teng et al. (2019) developed a method using 3D printing, MAO treatment and coprecipitation of Ca and P layers with BMP-2 technology to produce porous titanium alloy-based implants with interconnected channel structures. Growth factor BMP-2 continuously diffused from the central area to the peripheral area of the implant, which promoted the proliferation, differentiation and mineralization of bone cells. *In vivo* experiments showed that bone tissue and blood vessels grew into the central area of the implant. MAO-CaP-BMP-2 was superior to MAO and MAO-CaP groups in new bone formation, which indicates that MAO-CAP-BMP2 has the potential to promote bone healing. MAO-CaP-BMP2 is a good candidate for applications as a growth factor vector.

## Conclusions

There are broad opportunities in the fields of orthopedics and dentistry for the design of porous titanium-based alloy scaffolds and the combination of MAO with other surface modification technologies. However, several challenges must be resolved to accelerate their clinical implementation in medical implants. First, considering the complexity of bone structure, it is essential to optimize the characteristics of pore size, porosity and scaffold structure, and this will require numerous *in vitro* and *in vivo* tests. Second, appropriate processing methods for making porous materials should be explored. The disadvantages of using 3D printers to make porous materials are that it is not possible to completely melt powders and fully remove fine powders from pores in the materials. Moreover, the technological parameters of MAO are complicated, and optimal electrolyte composition and electrical parameters have not been determined; this will require many more experiments. At present, the osseointegration principle of MAO combined with other treatment methods is not clear and needs further exploration. Most of the controls implemented in commercial practice are based on experience and have significant potential for future development.
